# Some Common Causes of Pain in the Foot—II

**Published:** 1910-10-22

**Authors:** 


### Orthopædics.

### Some Common Causes of Pain in the Foot—II.

Apart from the pain which is the result of flat-foot, calf or leg pain may be caused by other deformities of the foot, chief among which are equino-varus and calcaneus. In both these conditions the pain may be severe, though it is never so constant or so wearying as in cases of flat-foot. The deformity must be corrected, and the correction maintained. Massage will be found of great use in relieving pains in these conditions.

### Metatarsalgia.

In this by no means uncommon condition the patient complains of intense lancinating neuralgic pain in the foot itself. The localisation of the pain is various; usually it is more complained of along the outer edge of the foot and over the third and fourth metatarsals; more rarely it is over the first metatarsal. This is the so-called Morton's disease, the exact pathology and causation of which are still obscure. It is usually stated to be very chronic, persistent and intractable, but in the majority of cases attention to the patient's footgear will work wonders in relieving the pain. Large, thin-soled, light boots or shoes must be worn, with plenty of room inside them to allow the patient to move his toes freely, and (a point of some importance) the boot should be so made as to allow the whole sole to rest flat on the ground when the patient stands erect and throws his full weight upon the arch. In other words, in the fully erect position the tips of the boot must be on the ground and not, as is generally the case in badly made boots, raised half an inch or more above the level of the heel. Care should be taken that proper socks are worn, light and not "ribbed," and some attention should be paid to the hygiene of the foot. Where the patient suffers from "perspiring feet," a common complication in this condition, the inside of the stockings may be dusted with a powder made up of equal parts of ground starch, zinc oxide, and salicylate of sodium. In bad cases it may be necessary to prepare a celluloid instep for use inside the shoe, with perforations over the tarsal bones. Most cases yield to this treatment, even such as have been defying ordinary treatment for many years. Here again, however, the instep must be moulded from an actual cast of the patient's foot, and must be applied by the medical attendant himself, who should carefully adapt each particular inset to the contour of the patient's foot. Such an inset must be frequently seen to and changed when it shows the least sign of weakness in any spot, as the perforations render it much less strong than the ordinary models used for flat-foot. Proper orthopædic treatment will usually serve to relieve most cases of Morton's disease, and only rarely will it be necessary to advise operative interference. Such interference is only justifiable when a probable cause for the metatarsalgia is found to exist in changes in the bones or tarsal joints, and can be demonstrated by the $x$-rays.

Korner of Munich has described a particular form of dorsal metatarsalgia affecting young subjects and leading to changes in the bones of the foot. Such cases are very rare, and are probably instances of early osteo-arthritic changes in the foot; but the condition is as yet obscure, and more information is wanted on the subject. The pain is said to be severe, and to yield readily to salicylates, from which it has been surmised that such cases are possibly rheumatic in origin.

### Osteo-arthritis.

In osteo-arthritis itself affecting the joints of the foot the pain is usually deeply seated, increased by commencing movements, but lessening after a few movements have been made, though as a rule it then rapidly increases in intensity. It is purely local, over the affected joint, and never felt in the leg. There is no superficial tenderness, while the objective signs of the causative lesion are usually apparent. It is a pain that is influenced by rest, and which does not increase at night or when the patient is in bed. The treatment is difficult, and the practitioner may have to try many means before he hits upon the one that suits the particular patient he is dealing with. The first trial should always be made with dry heat. A large piece of flannel is thoroughly warmed before the open fire and then wrapped round the whole foot. The flannel must be really hot, but care must be taken not to burn the patient's skin. This sometimes acts quickly and well, relieving the patient of all pain, and it is, moreover, a method which can be practised at home by the patient himself. Moist heat, by means of flannels wrung out in boiling water, is less satisfactory; cold, by ice bags or tubes, is practically useless. Massage and passive movement only appear to increase the pain in tarsal cases, however useful they are in cases where the hip, shoulder, or knee is affected. In obstinate cases a trial of radiant heat, $x$-rays, hot air, or electric-light baths may be made, and some cases which have resisted all other methods of treatment will be found to yield readily to one or other of these. A combination of dry heat with $x$-rays is very successful in some cases, the patients remaining free from pain for long intervals. Attention must, of course, be paid to relieving any pressure on the foot and correcting any deformity present, no matter what treatment is attempted. Internal medication is of little use. The continuous, weary, ache, so characteristic of arthritic disease, seems to be relieved by no drug that can be depended on. Potassium iodide, aspirin, guiacol (best given in the shape of the carbonate in capsules), the salicylates, and pyramidon appear to be among the best remedies to experiment with, but no reliance can be placed on any of them. A general tonic is often of marked benefit. Anodynes, such as morphia, must be strictly avoided. Counter-irritation by mustard plasters, cupping or pricking is absolutely useless, while the Bier method has been singularly unsuscessful in relieving pain in arthritic cases.

STRAINS AND SPRAINS.

These call for little attention apart from the injury itself. The pain usually yields readily to fomentations, though outside applications of opium lotion are no more efficacious in relieving it than a mere boracic solution properly applied. The dull aching pain that sometimes remains after a teno-synovitis is best treated by the hyperæmic method; a few applications of the bandage each day usually suffice to get rid of it in the course of a couple of days.

TUBERCULOUS DISEASE.

The pain in this condition is markedly severe, especially in senile cases. It is increased by warmth, and may rob the patient of all sleep, so that it is necessary to give injections of morphia to secure rest. The proper treatment here will, of course, depend on many points which must be carefully considered in each individual case. In advanced senile cases amputation is often the best treatment; it rids the patient of a useless and excessively painful foot which is not worth a trial to save. In younger patients and less advanced cases careful conservative treatment must be the rule, and here the Bier treatment is admirably suited to control the pain and promote the cure. The bandage should be used twice or thrice a day, being put on high above the knee, in cases where no sinuses exist. Where these are present the suction apparatus must be employed. The hyperæmic treatment is markedly efficacious in controlling the pain, but it must be borne in mind that on the first two or three applications of the bandage the patient may complain that the treatment gives him increased suffering. Tuberculin treatment, especially injections of the Baraneck's antitoxin, also improve the condition so far as the pain is concerned.

